# Transient Brugada Pattern Induced by Loperamide Abuse

**DOI:** 10.7759/cureus.8037

**Published:** 2020-05-09

**Authors:** Ali Atoot, Scott Sholem, Ibrahim Khaddash, Jamshed Zuberi

**Affiliations:** 1 Anesthesiology, Hackensack University Medical Center, Hackensack, USA; 2 Surgical Critical Care, Christiana Care Health System, Newark, USA; 3 Cardiology, St. Joseph's Regional Medical Center, Paterson, USA; 4 Surgery, St. Joseph's University Medical Center, Paterson, USA

**Keywords:** brugada syndrome, loperamide, loperamide toxicity, brugada pattern, drug toxicity

## Abstract

Brugada syndrome (BrS) is classically a malignant, genetically determined, arrhythmic syndrome manifesting as syncope or sudden cardiac death (SCD) in individuals with structurally normal hearts.^ ^An exceedingly rare cause of an induced electrocardiogram (ECG) pattern mimicking BrS is secondary to loperamide abuse. The following case describes the onset of a transient Brugada pattern secondary to loperamide abuse in a young healthy male.

## Introduction

Brugada syndrome (BrS) is classically a malignant, genetically determined, arrhythmic syndrome manifesting as syncope or sudden cardiac death (SCD) in individuals with structurally normal hearts. Loperamide abuse is an exceedingly rare cause of an induced electrocardiogram (ECG) pattern that mimics BrS. The following case describes the onset of a transient Brugada pattern secondary to loperamide abuse in a young healthy male. 

## Case presentation

A 28-year-old Caucasian male with a past medical history of asthma developed a sudden onset of diarrhea for which he started taking 2 mg of loperamide 10 times a day. He continued with this dosage for two weeks. After experiencing excruciating abdominal pain, he presented to a local ER for evaluation. Abdominal X-ray demonstrated an ileus. The emergency medicine physician believed the ileus to be caused by loperamide toxicity and recommended that the patient discontinue the loperamide immediately. After loperamide discontinuation the patient’s abdominal pain subsided, but he began to feel anxious and agitated. He attributed these symptoms to loperamide withdrawal and resumed taking his previous dosage. The symptoms persisted so he presented to his primary medical doctor, where he was found to have abnormal ECG findings. The patient was then sent to our institution for further workup.

**Figure 1:**
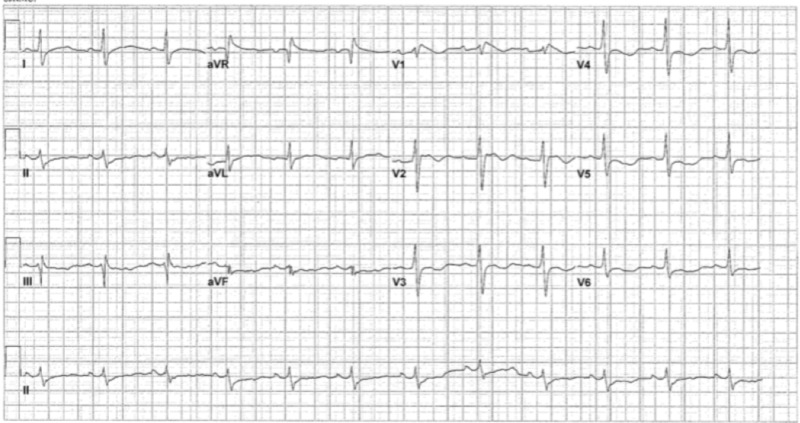
ECG showing signs of type 1 Brugada syndrome. ECG, electrocardiogram

In the ER, ECG demonstrated coved ST-segment elevations consistent with BrS (Figure 1).

A consult was placed to cardiology. The patient denied palpitations, syncope, near syncope, dizziness, chest pain, or shortness of breath. He reported previously abusing prescription medications, though denied any recent drug or alcohol use. The patient was not taking any medications apart from the loperamide. The patient was admitted to telemetry for cardiac monitoring. An echocardiogram demonstrated no regional wall motion abnormalities and left ventricular ejection fraction was preserved at 62%. On hospital day #2, the Brugada pattern on his ECG improved, and he was cleared for discharging home. The patient was counseled about loperamide abuse and was scheduled to follow-up outpatient with his cardiologist.

## Discussion

**Figure 2:**
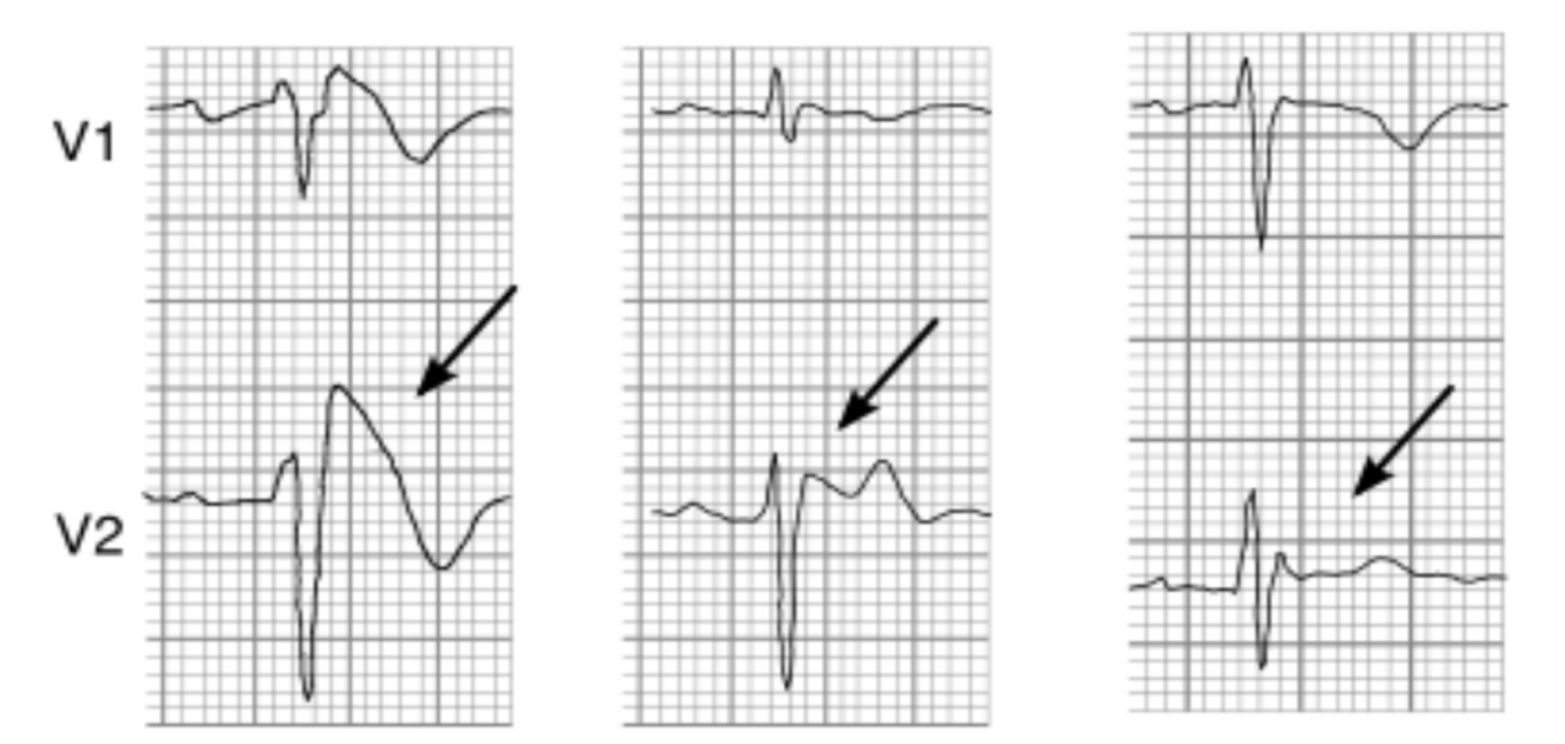
Three types of ST-segment elevation generally observed in patients with Brugada syndrome. The first tracing with coved type ST-segment elevation. The last two figures with saddle-back type ST-segment elevations.

Our patient presented with a transient Brugada pattern secondary to loperamide abuse. BrS is a malignant, genetically determined, arrhythmic syndrome manifesting as syncope or SCD in individuals with structurally normal hearts [1]. The ECG diagnostic pattern is characterized by coved ST-segment elevation in V1-V3 leads (Figure 2) [2-3].

The prevalence of BrS (0.01%-0.3%) varies among regions and ethnicities, with the highest prevalence in Southeast Asia. Presently, the majority of BrS patients are incidentally diagnosed, and may remain asymptomatic for their lifetime. However, BrS is responsible for 4%-12% of all SCDs and for ~20% of SCDs in patients with structurally normal hearts [4]. Several case series describe patients with cardiac conduction abnormalities and life-threatening ventricular arrhythmias temporally related to loperamide abuse [5]. However, upon reviewing the literature, very few cases have been described for a temporal relation of loperamide abuse and an ECG pattern mimicking BrS. 

Loperamide, a nonprescription anti-diarrheal agent, is a peripheral mu-opioid receptor agonist that is excluded from the blood-brain barrier by p-glycoprotein at therapeutic doses. Loperamide produces both QRS and QT prolongation at supra-therapeutic dosing [6]. BrS patients can sometimes have normal ECG recordings. In these cases, a diagnostic challenge with a sodium channel blocker such as ajmaline, flecainide, or pilsicainide may induce the full-blown Brugada ECG pattern and support the diagnosis [7]. However, many other pharmacologic agents not related to class I anti-arrhythmic agents have been reported to induce Brugada ECG patterns including tricyclic antidepressants, fluoxetine, lithium, trifluoperazine, antihistamines, and cocaine [7]. As more published reports of loperamide and other drug-induced Brugada patterns increases, there is greater interest as to the mechanism responsible for these occurrences. These patterns may be due to an individual susceptibility that favors drug-induced ECG abnormalities, such as an increase in latent ion channel dysfunction similar to drug-induced long QT syndrome [7]. Further research needs to be performed to investigate the populations at risk for these type of cardiac events.

## Conclusions

Toxicity related to loperamide, an opioid agonist that is readily available without a prescription occurs with moderate frequency. Loperamide is used in patients with opioid dependence to treat diarrhea secondary to withdrawal. However, it can be used to mimic opioid effects secondary to mu-receptor activation. It is important for clinicians to be aware of the potentially life-threatening cardiac toxicity related to loperamide abuse in order to provide prompt diagnosis, management, and patient education.
